# Identification of hub genes and their expression profiling for predicting buffalo (*Bubalus bubalis*) semen quality and fertility

**DOI:** 10.1038/s41598-023-48925-5

**Published:** 2023-12-13

**Authors:** Divakar Swathi, Laxman Ramya, Santhanahalli Siddalingappa Archana, Balaganur Krishnappa, Bala Krishnan Binsila, Sellappan Selvaraju

**Affiliations:** 1 Reproductive Physiology Laboratory, Animal Physiology Division, ICAR-National Institute of Animal Nutrition and Physiology, Adugodi, Bengaluru, 560030 India; 2 Department of Biotechnology, Jain University, Bengaluru, 560001 India

**Keywords:** Transcriptomics, Molecular medicine, Predictive markers

## Abstract

Sperm transcriptomics provide insights into subtle differences in sperm fertilization competence. For predicting the success of complex traits like male fertility, identification of hub genes involved in various sperm functions are essential. The bulls from the transcriptome profiled samples (n = 21), were grouped into good and poor progressive motility (PM), acrosome integrity (AI), functional membrane integrity (FMI) and fertility rate (FR) groups. The up-regulated genes identified in each group were 87, 470, 1715 and 36, respectively. Gene networks were constructed using up- and down-regulated genes from each group. The top clusters from the upregulated gene networks of the PM, AI, FMI and FR groups were involved in tyrosine kinase (FDR = 1.61E−11), apoptosis (FDR = 1.65E−8), translation (FDR = 2.2E−16) and ribosomal pathway (FDR = 1.98E−21), respectively. From the clusters, the hub genes were identified and validated in a fresh set of semen samples (n = 12) using RT-qPCR. Importantly, the genes (fold change) *RPL36AL* (14.99) in AI, *EIF5A* (54.32) in FMI, and *RPLP0* (8.55) and *RPS28* (13.42) in FR were significantly (p < 0.05) up-regulated. The study suggests that the expression levels of *MAPK3* (PM), *RPL36AL* + *RPS27A* or *RPL36AL* + *EXT2* (AI), *RPL36AL* or *RPS27A* (FMI) and *RPS18* + *RPS28* (FR) are potential markers for diagnosing the semen quality and fertility status of bulls which can be used for the breeding program.

## Introduction

In the dairy industry, bulls are selected for artificial insemination (AI) programs based on breeding soundness evaluations and conventional tests that measure sperm functions^1^. Conventional tests to establish fertility differences among bulls are inadequate because the results are highly variable and do not correlate with bull fertility status^2^. Measures for the selection of high-fertile and high-quality semen producing bulls are of utmost importance as they bring immense economic benefits to frozen semen stations and farmers. Hence, next-generation omics technologies are in high demand to identify subtle variations in molecular signatures that influence sperm quality, which ultimately determines the fertility status of bulls. Recently, sperm transcriptomic and proteomic profiling have identified the factors and pathways associated with semen quality and fertility rate (FR) in human^3^ and bovine^1,4^. Such detailed information on Murrah buffalo bulls is not currently available.

Importantly, fertilization is a multifaceted phenomenon involving numerous associated sub-processes or factors. Sperm progressive forward motility is an indispensable trait for travelling across various barriers at different segments of the cervix, uterus and oviduct. In addition, the functional membrane integrity of sperm is an essential attribute for combating the biochemical micro-milieu of the female reproductive tract. Acrosomal membrane intactness is a prerequisite for sperm capacitation in the uterus and acrosomal reaction in the oviduct. All these sperm attributes are essential components of effective fertilization and required to identify high-fertile bulls for breeding program.

Distinctly, any perturbation in these biological processes is the consequence of a disturbance in a group of genes and not from a single gene alone^5^. Although omics technologies generate big data, the process of extracting the desired information from them becomes challenging. Moreover, advancements in transcriptome data analysis have led to the establishment of “gene regulatory networks” and which comprise coordinately expressed genes. Hub genes in the network tend to cluster densely and represent the likely control points of the study condition^6,7^. Studies have established that hub genes can serve as diagnostic, predictive, or prognostic biomarkers in colon adenocarcinoma^8^. In bovines, hub genes involved in mastitis development^9^, intramuscular fat content of Nellore cattle^10^ and in vivo pre-implantation development^11^ are identified. However, such information for male fertility prediction is not available for any species.

Since effective fertilization involves diversified sperm features, the identification of hub genes for fertility prediction may aid in accurately diagnosing the fertility status of bulls. Furthermore, the identified hub genes involved in sperm function and fertility may provide new insights for developing guidelines for bull fertility assessment.

In the present study, sperm transcriptome data available in the laboratory from Murrah buffalo bulls were used retrospectively to identify the differentially expressed genes in the progressive motility (PM), acrosomal integrity (AI), functional membrane integrity (FMI) and FR groups. These differentially expressed genes can be used to identify hub genes that regulate sperm functions and FR. The present study aimed to (1) identify the hub genes involved in the regulation of sperm functions and fertility and (2) evaluate the fertility prediction ability of the identified hub genes using receiver operating characteristic (ROC) curve analysis.

## Results

### Grouping of bulls based on sperm functions and fertility rate

**Figure 1:**
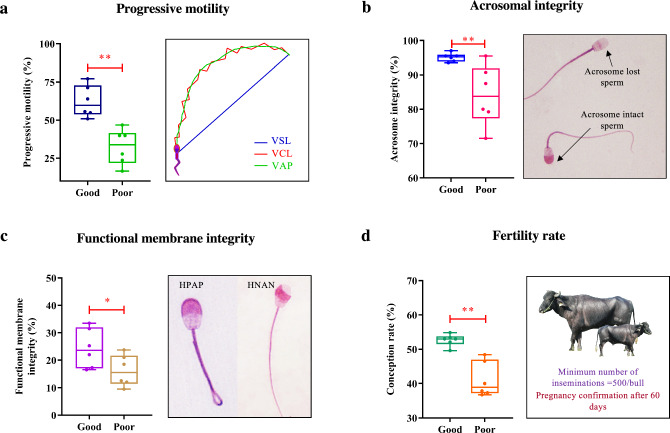
Grouping of animals based on sperm functions and fertility rate. Sperm functions, such as progressive motility (**a**), acrosomal integrity (**b**), functional membrane integrity (**c**) and fertility rate (**d**) were higher in good as compared to poor semen-producing groups. ** denotes significant difference between the groups (*p* < 0.01); * denotes *p* < 0.05.

Bulls (n = 12) were classified into two groups (good and poor semen quality producers) based on the group averages for sperm functions and FR. The sperm functions and fertility rates were significantly (*p* < 0.05) differing between the respective good and poor groups of PM (62.31 ± 4.22 vs 32.44 ± 4.72), AI (92.86 ± 0.88 vs 83.02 ± 1.81), FMI (28.66 ± 2.00 vs 13.81 ± 1.34) and FR (52.67 ± 0.72 vs 41.16 ± 2.05) (Fig. 1a–d).

### Differentially expressed genes from the transcriptome library

**Figure 2:**
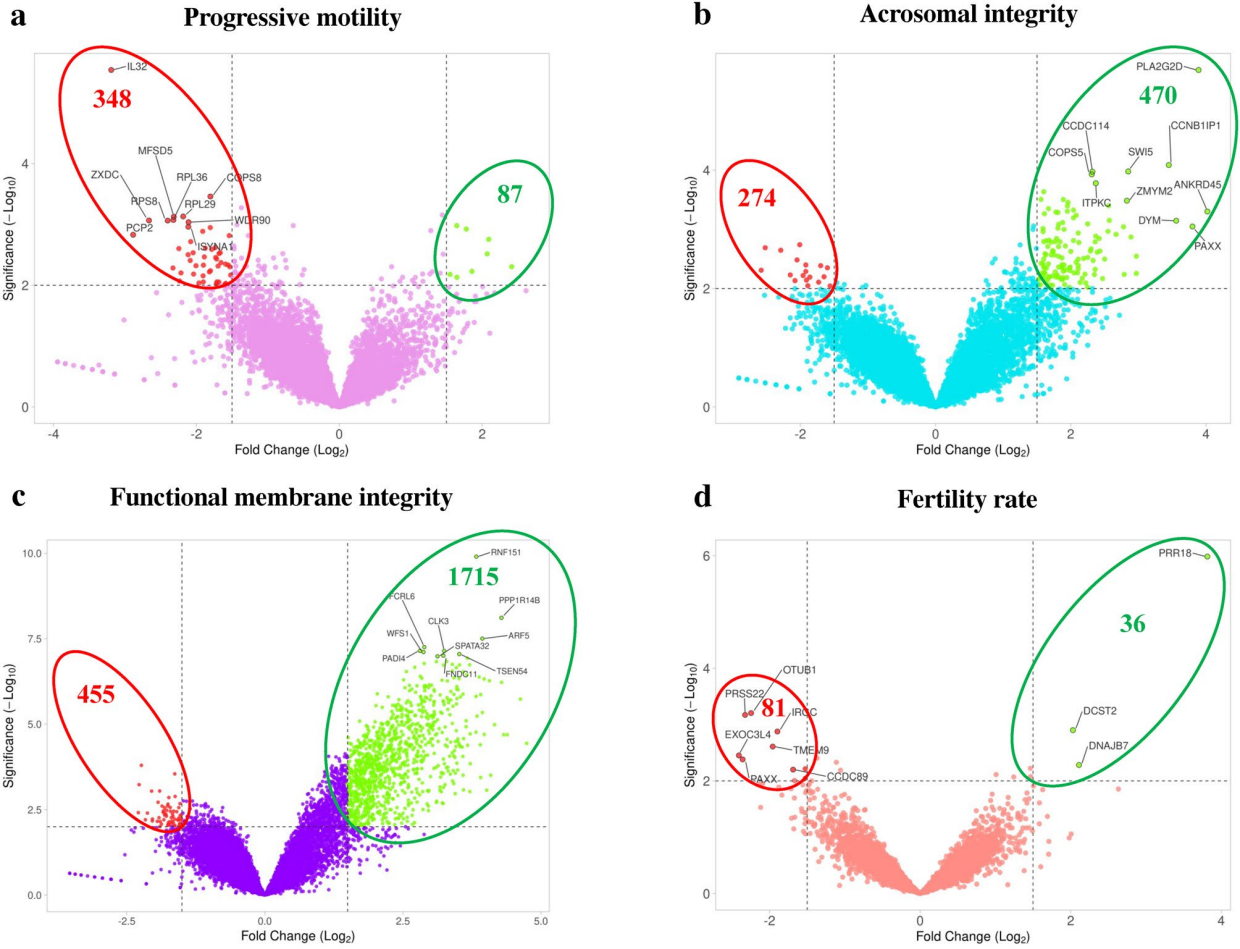
Volcano plots of the differentially expressed genes in each group. The genes from the transcriptome data having log_2_ fold change > ± 1 and *p* < 0.05 were considered as differentially expressed genes. There were 435, 744, 2170 and 117 genes differentially expressed in the progressive motility (**a**), acrosome integrity (**b**), functional membrane integrity (**c**) and fertility rate (**d**) groups, respectively.

Differentially expressed genes with a predefined threshold of log_2_FC greater than 1.0 and *p* < 0.05 in the PM, AI, FMI and FR groups were identified (Fig. 2a–d). There were 87, 470, 1715 and 36 genes up-regulated (Table 1) and 348, 274, 455 and 81 genes down-regulated in PM, AI, FMI, and FR groups, respectively (Table 2).

**Table 1:** List of up-regulated genes from sperm functions and fertility groups. Significant and abundantly expressed top 10 genes from progressive motility, acrosome integrity, functional membrane integrity and fertility rate groups and their functions were reported. *PAXX* gene was commonly found in the top 10 genes of the two groups.

S. no.	Genes and gene description	log2 fold change	*p*-value	Gene function
(a) Progressive motility				
1	PAXX non-homologous end joining factor *(PAXX)*	2.61	1.23E−02	Cellular response to DNA damage stimulus
2	Caspase 6 *(CASP6)*	2.41	4.96E−03	Protein auto processing
3	G protein-coupled receptor class C group 6 member A *(GPRC6A)*	2.18	1.33E−02	G-protein coupled receptor signaling pathway
4	Immunoglobulin superfamily member 23 *(IGSF23)*	2.09	1.76E−03	Immune response pathway
5	UL16 binding protein 3 *(ULBP3)*	2.07	3.05E−03	Natural killer cell activation
6	Cell death inducing DFFA like effector A *(CIDEA)*	2.06	1.39E−02	Apoptotic process
7	WASH complex subunit 1 *(WASCH1)*	2.01	1.05E−02	Protein transport
8	Family with sequence similarity 24 member A *(FAM24A)*	1.90	1.51E−02	Outer dynein arm assembly
9	Nuclear receptor coactivator 4 *(NCOA4)*	1.89	1.86E−02	Protein targeting to lysosome
10	Transmembrane protein 225 *(TMEM225)*	1.89	4.08E−02	Negative regulation of phosphatase activity
(b) Acrosome integrity				
1	Ankyrin repeat domain 45 *(ANKRD45)*	4.02	4.99E−04	Cell proliferation
2	Phospholipase A2 group IID *(PLA2G2D)*	3.89	2.03E−06	CD4-positive, CD25-positive, alpha–beta regulatory T cell differentiation
3	PAXX non-homologous end joining factor *(PAXX)*	3.80	8.98E−04	Cellular response to DNA damage stimulus
4	Dymeclin *(DYM)*	3.56	7.16E−04	Golgi organization
5	Cyclin B1 interacting protein 1 *(CCNB1IP1)*	3.45	8.21E−05	Chiasma assembly
6	Transition protein 2 *(TNP2)*	2.97	5.57E−03	Positive regulation of protein processing
7	G protein-coupled receptor class C group 6 member A *(GPRC6A)*	2.97	2.85E−03	G-protein coupled receptor signaling pathway
8	Caspase 6 *(CASP6)*	2.89	1.35E−03	Protein auto-processing
9	SWI5 homologous recombination repair protein *(SWI5)*	2.85	1.05E−04	Cellular response to ionizing radiation
10	Zinc finger MYM-type containing 2 *(ZMYM2)*	2.83	3.28E−04	Negative regulation of transcription, DNA-templated
(c) Functional membrane integrity				
1	Cerebellar degeneration related 1 *(CDR1)*	4.74	3.65E−05	Regulation of salicylic acid metabolic process
2	Tumor suppressor 2, mitochondrial calcium regulator *(TUSC2)*	4.63	1.85E−06	Phagocytosis
3	PAXX non-homologous end joining factor *(PAXX)*	4.39	2.04E−05	Cellular response to DNA damage stimulus
4	Chromosome 11 C14orf119 homolog *(C11H14orf119)*	4.33	2.03E−05	Chromosomal homolog
5	DExD/H-box helicase 60 *(DDX60)*	4.29	5.99E−07	Positive regulation of RIG-I signaling pathway
6	Protein phosphatase 1 regulatory inhibitor subunit 14B *(PPP1R14B)*	4.29	7.70E−09	Regulation of phosphorylation
7	Immunoglobulin superfamily member 23 (IGSF23)	4.16	1.03E−05	Immune response pathway
8	Kelch like family member 25 *(KLHL25)*	4.09	3.12E−04	Positive regulation of CD4-positive, CD25-positive, alpha–beta regulatory T cell differentiation
9	ATP synthase membrane subunit E *(ATP5ME)*	4.07	5.38E−07	ATP synthesis coupled proton transport
10	Fucosyltransferase 8 *(FUT8)*	4.02	8.56E−04	N-glycan fucosylation
(d) Fertility rate				
1	Proline rich 18 *(PRR18)*	3.82	1.04E−06	Paralog of reticulon
2	Coiled-coil domain containing 169 *(CCDC169)*	2.63	1.39E−02	Involved in hedgehog signalling
3	DNAJ heat shock protein family (Hsp40) member B7 *(DNAJB7)*	2.11	5.22E−03	Chaperone-mediated protein folding
4	DC-STAMP domain containing 2 *(DCST2)*	2.03	1.25E−03	Sperm-egg recognition
5	Ribosomal protein S4 X-linked *(RPS4X)*	1.69	4.27E−02	Cytoplasmic translation
6	G protein-coupled receptor 19 *(GPR19)*	1.63	1.43E−02	G-protein coupled receptor signaling pathway
7	Thiosulfate sulfurtransferase like domain containing 3 *(TSTD3)*	1.58	2.46E−02	Pumilio like repeats
8	Epoxide hydrolase 4* (EPHX4)*	1.51	1.23E−02	Lipid metabolic process
9	Calicin *(CCIN)*	1.47	8.39E−03	Cell differentiation
10	Epididymal protein 13 *(EDDM13)*	1.46	5.98E−03	Integral component of a membrane

**Table 2:** List of down-regulated genes from sperm functions and fertility groups. Significant and abundantly expressed top 10 down-regulated genes, their description, log_2_fold change, p-value and gene function from each group were reported.

S. no.	Genes and gene description	log2 Fold change	*p-*value	Gene function
(a) Progressive motility				
1	Interleukin 32 *(IL32)*	− 3.19	2.89E−06	Immune response
2	Ring finger protein 224 *(RNF224)*	− 3.01	3.47E−04	Metal ion binding
3	Purkinje cell protein 2 *(PCP2)*	− 2.89	5.32E−04	Killing of cells of another organism
4	ZXD family zinc finger C *(ZXDC)*	− 2.66	7.39E−04	Positive regulation of transcription, DNA-templated
5	SS nuclear autoantigen 1 *(SSNA1)*	− 2.56	7.47E−04	Axon extension
6	Ribosomal protein S8 *(RPS8)*	− 2.40	8.46E−04	Cytoplasmic translation
7	Insulin *(INS)*	− 2.39	8.59E−04	Positive regulation of cell proliferation
8	Leucine rich repeat containing 14 *(LRRC14)*	− 2.33	8.65E−04	Negative regulation of NF-kappaB transcription factor activity
9	Ribosomal protein L36 *(RPL36)*	− 2.32	9.18E−04	Cytoplasmic translation
10	Major facilitator superfamily domain containing 5 *(MFSD5)*	− 2.32	1.01E−03	Molybdate ion transmembrane transport
(b) Acrosome integrity				
1	Small regulatory polypeptide of amino acid response *(SPAAR)*	− 2.58	4.91E−03	Cellular response to amino acid stimulus
2	Mitochondrial elongation factor 2 *(MIEF2)*	− 2.53	1.30E−02	Mitochondrion organization
3	Vascular endothelial growth factor B *(VEGFB)*	− 2.52	2.05E−03	Vascular endothelial growth factor receptor signaling pathway
4	Achaete-scute family bHLH transcription factor 2 *(ASCL2)*	− 2.32	1.39E−02	Somatic stem cell population maintenance
5	Major facilitator superfamily domain containing 5 *(MFSD5)*	− 2.29	2.26E−03	Molybdate ion transport
6	Transmembrane epididymal protein 1 *(TEDDM1)*	− 2.22	4.70E−02	Integral component of membrane
7	Translocase of inner mitochondrial membrane 13 *(TIMM13)*	− 2.15	3.14E−02	Sensory perception of sound
8	Single Ig and TIR domain containing *(SIGIRR)*	− 2.15	5.90E−03	Signal transduction
9	Homeobox D9 *(HOXD9)*	− 2.13	1.56E−02	Transcription, DNA-templated
10	RecQ like helicase 4 *(RECQL4)*	− 2.12	1.36E−02	Telomere maintenance
(c) Functional membrane integrity				
1	Fumarylacetoacetate hydrolase domain containing 1 *(FAHD1)*	− 2.42	1.25E−02	Pyruvate metabolic process
2	SRY-box transcription factor 1 *(SOX1)*	− 2.37	1.44E−02	Negative regulation of transcription from RNA polymerase II promoter
3	Small integral membrane protein 10 *(SMIM10)*	− 2.35	1.09E−02	Integral component of membrane
4	Transketolase like 2 *(TKTL2)*	− 2.29	3.87E−03	Total Transketolase activity
5	Orthodenticle homeobox 1 *(OTX1)*	− 2.28	5.89E−04	Metencephalon development
6	Prostaglandin I2 receptor *(PTGIR)*	− 2.23	1.61E−04	Negative regulation of smooth muscle cell proliferation
7	Taste 2 receptor member 16 *(TAS2R16)*	− 2.20	1.11E−02	G-protein coupled receptor signaling pathway
8	Myogenic differentiation 1 *(MYOD1)*	− 2.16	6.25E−03	Regulation of alternative mRNA splicing, via spliceosome
9	TIMP metallopeptidase inhibitor 1 *(TIMP1)*	− 2.13	4.00E−03	Negative regulation of endopeptidase activity
10	Poly(A) binding protein cytoplasmic 4 like *(PABPC4L)*	− 2.05	9.09E−04	RNA Binding activity
(d) Fertility rate				
1	Exocyst complex component 3 like 4 *(EXOC3L)*	− 2.41	3.53E−03	Exocytosis
2	PAXX non-homologous end joining factor *(PAXX)*	− 2.36	4.16E− 03	Cellular response to DNA damage stimulus
3	Serine protease 22 *(PRSS22)*	− 2.32	6.73E−04	Positive regulation of peptidase activity
4	OTU deubiquitinase, ubiquitin aldehyde binding 1 *(OTUB1)*	− 2.25	6.24E−04	Adaptive immune response
5	Peroxiredoxin like 2b *(FAM213B)*	− 2.14	1.15E−02	Prostaglandin biosynthetic process
6	Glutathione peroxidase 4 *(GPX4)*	− 2.12	2.95E−02	Response to oxidative stress
7	ATP synthase membrane subunit E *(ATP5ME)*	− 2.09	1.13E−02	ATP synthesis coupled proton transport
8	Transmembrane protein 9 *(TMEM9)*	− 1.96	2.46E−03	Lysosomal lumen acidification
9	Immunity related GTPase cinema *(IRGC)*	− 1.90	1.33E−03	Defense response
10	Serine/threonine kinase 25 *(STK25)*	− 1.88	1.29E−02	Establishment of Golgi localization

### Gene-set enrichment analysis of the up-regulated genes in sperm functions and fertility rate

**Figure 3:**
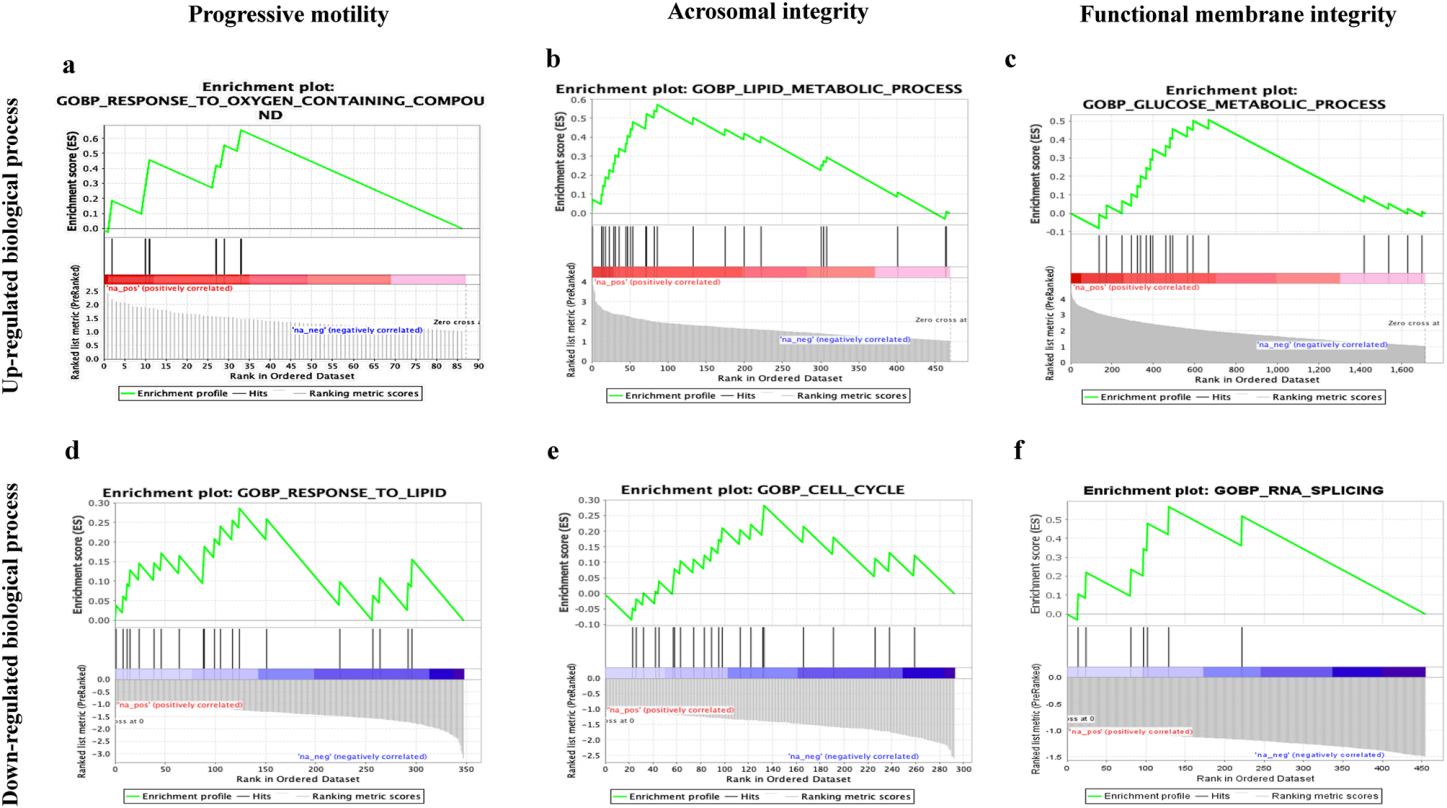
Gene-set enrichment analysis of the differentially expressed genes. The enriched biological processes of the up-regulated and down-regulated genes were response to oxygen containing compound (**a**) and regulation of anatomical structure morphogenesis (**b**) in PM, lipid metabolic process (**c**) and cell cycle (**d**) in AI, glucose metabolic process (**e**) and RNA splicing (**f**) in FMI. There was no significant enrichment in the differentially expressed genes of the fertility rate group.

Gene-set enrichment analysis of the up-regulated genes revealed that biological processes such as response to oxygen-containing compounds (Normalized Expression Score: NES = 1.79), lipid metabolic processes (NES = 2.62) and defense responses (NES = 1.67), glucose metabolic processes (NES = 1.90) and fertilization (NES = 1.68) were enriched in the up-regulated genes of good PM, AI, and FMI, respectively (Table 3; Fig. 3a–c). In the FR group, there was no significantly enriched biological process.

**Table 3:** List of significantly enriched biological processes of the up-regulated genes in each group. There were two, six and 22 biological processes enriched in progressive motility, acrosomal integrity and functional membrane integrity, respectively (The redundant processes were not given in the table). No significant enrichment was found in up-regulated genes of the fertility rate group. (*NES*: Normalized Enrichment Score).

Enriched biological processes	NES	*p-*value	Genes involved
(a) Progressive motility			
Response to oxygen containing compound	1.79	0.01	*GPRC6A, NCOA4, IL36G*
(b) Acrosome integrity			
Lipid metabolic process	2.62	0.00	*PLA2G2D, WASHC1, GDE1*
Lipid biosynthetic process	1.84	0.02	*IGFBP7, LIAS, PIGM*
Defense response to another organism	1.69	0.03	*CASP6, AKAP1, DDX60*
Defense response	1.67	0.03	*PLA2G2D, CASP6, AKAP1*
Biological process involved in interspecies interaction between organisms	1.65	0.02	*CASP6, AKAP1, LIAS*
(c) Functional membrane integrity			
Glucose metabolic process	1.90	0.00	*ADPGK, MIDN, GAPDHS*
Defense response to symbiont	1.84	0.00	*DDX60, IL21, IL4*
Regulation of cellular macromolecule biosynthetic process	1.69	0.00	*KLHL25, CD28, JAK3*
Monosaccharide metabolic process	1.68	0.01	*FUT8, ADPGK, MIDN*
Response to virus	1.67	0.02	*DDX60, IL21, IL4*
Protein maturation	1.67	0.02	*LIAS, CASP6, DNAJB11*
Fertilization	1.68	0.02	*SPESP1, TSSK4, TNP2*
Energy derivation by oxidation of organic compounds	1.69	0.02	*ATP5ME, ATP5F1D, PPP1R3E*
Amide biosynthetic process	1.50	0.03	*KLHL25, RPL13, MRPS7*
Regulation of carbohydrate metabolic process	1.61	0.03	*MIDN, GAPDHS, PPP1R3E*
CD4 positive alpha beta T cell activation	1.58	0.03	*CD80, IL21, PLA2G2D*
Lipid biosynthetic process	1.49	0.04	*IGFBP7, PIGL, ARMC5*
Carbohydrate biosynthetic process	1.56	0.04	*FUT8, B4GAT1, PPP1R3E*
Positive regulation of cytokine production	1.50	0.04	*KLHL25, NCK2, CD28*
Alpha beta T cell activation	1.62	0.04	*CD80, IL21, CD28*
Organophosphate biosynthetic process	1.52	0.04	*ATP5ME, NME4, NUDT2*
Regulation of cellular carbohydrate metabolic process	1.53	0.05	*MIDN, PPP1R3E, PPP1CA*
(d) Fertility rate			
No significant enrichment

The cellular components microtubule organizing center (NES = 1.60) in PM, endosome (NES = 1.42), plasma membrane (NES = 1.3) and acrosomal vesicle (NES = 1.90) in AI were enriched in the up-regulated genes. Interestingly, the up-regulated genes of both FMI and FR were enriched in the ribonucleoprotein complex (FMI: NES = 1.43 and FR: NES = 0.51).

Molecular functions, such as cytoskeletal protein binding (NES = 1.47) and zinc ion binding (NES = 1.70), were enriched in PM and AI, respectively. In contrast, protein serine kinase activity (NES = 1.76), catalytic activity against RNA (NES = 1.63) and phosphatase binding (NES = 1.65) were enriched in FMI group.

In the down-regulated genes, response to lipid (NES = 1.58), cell cycle (NES = 1.62) and membrane organization (NES = 1.62) were the enriched biological processes in PM, AI, and FMI, respectively (Fig. 3d–f) and they were localized at cell surface (NES = 2.08) and catalytic complex (NES = 2.17) in the AI and FMI groups, respectively. Likewise, enzyme regulator activity (NES = 1.73) and zinc ion binding (NES = 1.73) were the enriched molecular functions in AI and FMI, respectively. There was no significant enrichment in the FR group.

### Gene interaction network of the sperm functions and fertility rate

The network of the up-regulated genes consisted of 90 and 121, 248 and 399, 1096 and 4779, and 44 and 91 nodes and edges corresponding to the PM, AI, FMI, and FR groups, respectively. Subsequently, merging of the four networks revealed 1736 nodes and 8787 edges. The intersection of all four networks resulted in single node *EXT2* (Supplementary Fig. S1). This gene was considered along with hub genes for RT-qPCR validation and fertility prediction model analysis.

Similarly, the networks of down-regulated genes had 354 and 992, 293 and 605, 590 and 1662, and 90 and 166 nodes and edges in the PM, AI, FMI, and FR groups, respectively (Supplementary Fig. S2). However, there was no intersecting down-regulated gene for all the four groups.

### Identification of the hub genes in sperm functions and fertility rate

The clusters or interconnected sub-networks obtained from each group (Table 4) indicated that the number of clusters and the cluster score were dependent on the number of up-regulated genes used for the analysis.

**Table 4:** Cluster analysis of significantly upregulated genes in sperm. Cluster analysis was performed using the upregulated genes in progressive motility, acrosomal integrity, functional membrane integrity and fertility rate using MCODE. The groups with high numbers of up-regulated genes had more clusters, which in turn had many nodes and edges.

S. no.	Group	Number of clusters	Cluster number	Number of nodes or genes	Number of edges
1	Progressive motility	1	Cluster 1	21	89
2	Acrosomal integrity	3	Cluster 1	17	107
			Cluster 2	3	3
			Cluster 3	3	3
3	Functional membrane integrity	36	Cluster 1	24	253
			Cluster 2	46	173
			Cluster 3	13	39
			Cluster 4	23	67
			Cluster 5	95	277
4	Fertility rate	1	Cluster 1	15	88

In the up-regulated gene network, the top cluster in the PM had 13 nodes and 58 edges, with a cluster score of 9.67. They were involved in the enzyme-linked receptor protein signaling process (FDR = 5.04E−10) and tyrosine kinase pathways (FDR = 1.61E−11). In the AI group, the top cluster had 17 nodes and 107 edges with a cluster score of 13.375. These nodes were involved in intracellular signal transduction (FDR = 1.2E−11) and apoptosis pathways (FDR = 1.65E−8). Furthermore, in the FMI, 29 nodes and 235 edges with a cluster score of 16.786 were observed. These genes were involved in protein localization to the membrane (FDR = 1.11E−13) and translation pathways (FDR = 2.2E−16). Finally, in FR, 13 nodes and 73 edges were observed with a score of 12.167. They were associated with translation (FDR = 5.68E−17) and ribosomal pathway (FDR = 1.98E−21) processes.

Likewise, in the down-regulated genes, the top cluster of PM group had 22 nodes and 103 edges with a cluster score of 9.81 and the genes were involved in amide biosynthetic process (FDR = 2.07E−7) and translation (FDR = 2.68E−7) processes. In the AI group, the top cluster had 21 nodes with 178 edges with a cluster score of 17.8 and they correspond to positive regulation of cell population proliferation (FDR = 1.03E−8) and positive regulation of gene expression (FDR = 1.03E−8) processes. Similarly, the top cluster of FMI group had 14 nodes and 89 clusters with the cluster score of 19.586 and associated with regulation of cell population proliferation (FDR = 2.98E−15) and cell surface receptor signaling pathway (FDR = 2.21E−12). In the FR group, the top cluster had 14 nodes, 89 edges and cluster score of 13.692 with the functional enrichment of translation (FDR = 7.72E−18) and SRP-dependent co-translational protein targeting to membrane (FDR = 4.42E−17).

**Figure 4:**
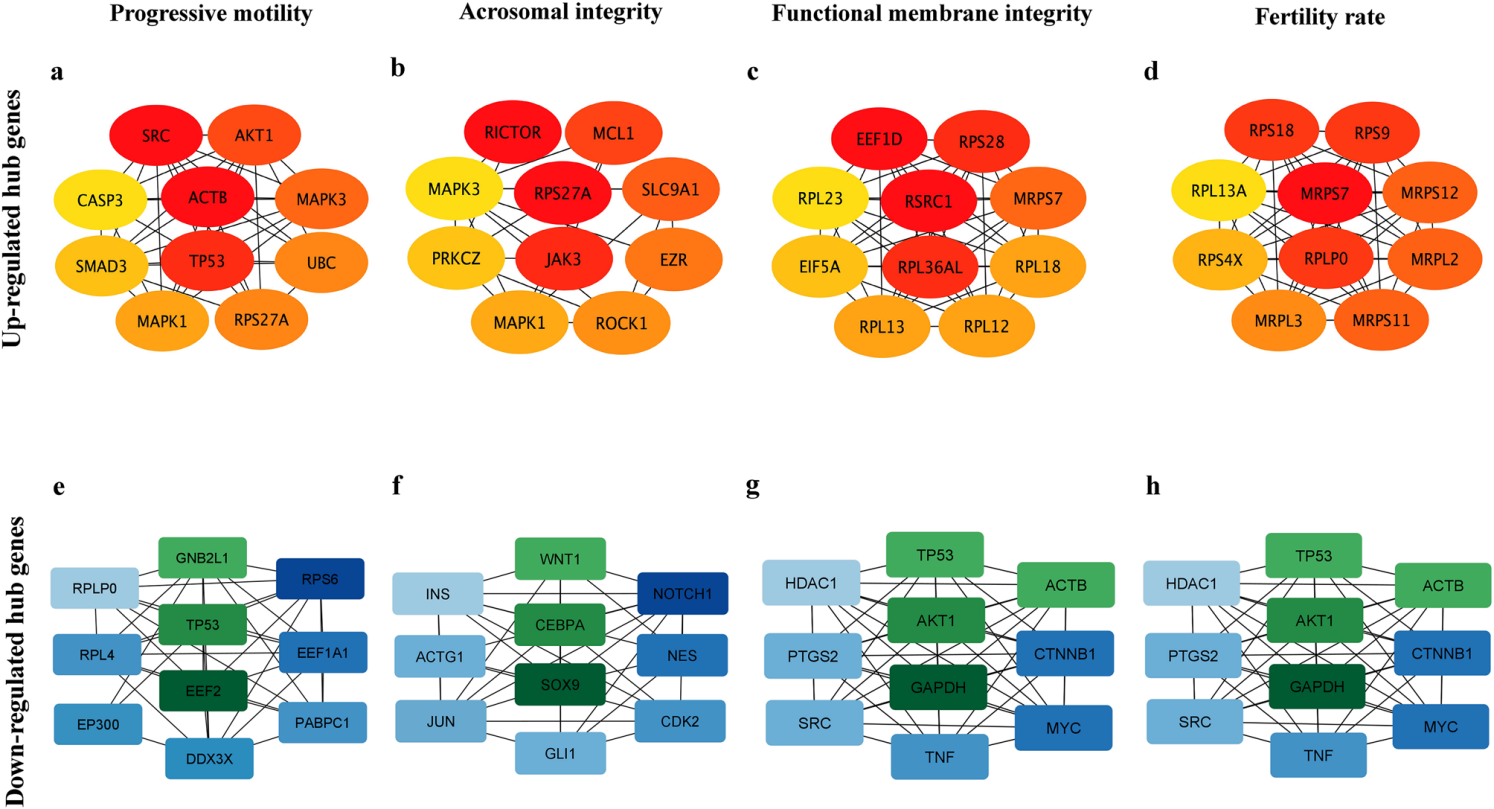
Hub genes identified in each of the four groups. The hub genes of both the up and down-regulated genes were identified in each of the groups progressive motility (**a**,**e**), acrosomal integrity (**b**,**f**), functional membrane integrity (**c**,**g**) and fertility rate (**d**,**h**). These nodes were identified from the intersection of all the analysis methods given in the Cytohubba. Variations in the colour from red to yellow represent more to less likely control points. Edges represent the association between the nodes.

From these clusters, the top ten up-regulated hub genes were identified in each group. Ribosomal protein gene families (both ribosomal protein large (RPL) and ribosomal protein small (RPS) subunits) were common to all four groups (Fig. 4a–d). These results indicate a probable role of sperm-retained ribosomal transcripts in fertility regulation. Similarly, the down-regulated hub genes of the PM and FR group had ribosomal protein gene families with the enrichment of translation process (Fig. 4e,h). Whereas the AI and FMI group had hub genes enriched in the regulation of response to stimulus, and regulation of proteolysis processes, respectively (Fig. 4f,g).

### Validation of the expression levels of hub genes and overlapping gene

**Figure 5:**
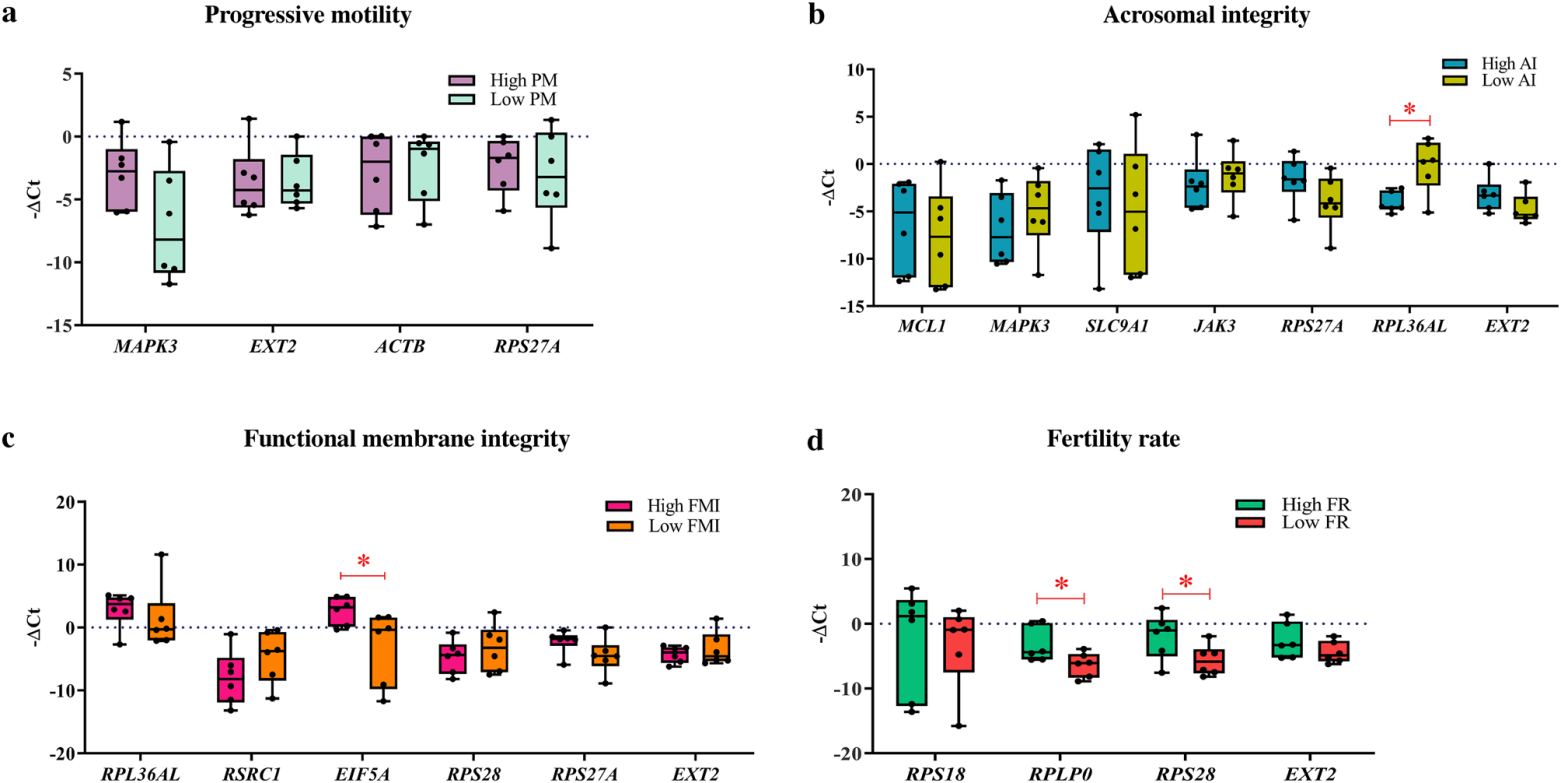
Validation of the hub genes using RT-qPCR. The hub genes in each of the groups progressive motility (**a**), acrosomal integrity (**b**), functional membrane integrity (**c**), the fertility rate (**d**) and the overlapping gene *EXT2* were validated using *GAPDHS* as a housekeeping gene. The hub genes *RPL36AL*, *EIF5A,* *RPLP0* and *RPS28* were significantly (*p* < 0.05) differentially expressed.

The genes (fold change) *MAPK3* (17.10) and *RPS27A* (1.80) in PM; *MCL1* (2.38), *SLC9A1* (2.73), *RPS27A* (5.22), *RPL36AL* (14.99) and *EXT2* (2.89) in AI; *RPL36AL* (2.81), *EIF5A* (54.32) and *RPS27A* (4.78) in FMI; and *RPS18* (1.70)*, RPLP0* (8.55)*, RPS28* (13.42) and *EXT2* (3.50) in FR were up-regulated. Among these genes, *RPL36AL* in AI, *EIF5A* in FMI and *RPLP0* and *RPS28* in the FR group were significantly (*p* < 0.05) differentially expressed (Fig. 5a–d). Genes influencing PM (*ACTB*)*,* AI (*MAPK3*) and (*JAK3*) as well as the FMI gene (*RSRC1*) were down-regulated in their corresponding good-quality semen-producing bulls. The overlapping gene *EXT2* was up-regulated in the AI and FR groups and hence, they were included in those groups for assessing sperm functions and FR predictability. The representative proteins expression levels of RPS18, RPLP0 and EXT2 showed that only EXT2 significantly higher in low fertility group as compared to high fertility group (Supplementary Fig. S3).

### Influence of hub genes on sperm functions and fertility rate

The RT-qPCR expression levels of the hub genes were strongly correlated with sperm functions and fertility rate (Table 5). Importantly, *RPLP0* (*r* = 0.58) and *EIF5A* (*r* = 0.62) were significantly (*p* < 0.05) correlated with FR and FMI, respectively. Interestingly, the gene *RPL36AL* (*r* = 0.73) was strongly correlated with AI rather than FMI.

**Table 5:** Correlation matrix between the expression levels of hub genes with sperm functions and fertility rate in buffalo. Though the expression levels of all the studied genes had a positive influence, the genes such as *RPL36AL, EIF5A* and *RPLP0* had significant correlation with acrosome integrity, functional membrane integrity and fertility rate, respectively. (*p<0.05; **p<0.01)

Hub genes	Progressive motility	Acrosome integrity	Functional membrane integrity	Fertility rate
*RPLP0*	0.34		0.35	0.58*
*RPS28*		0.33		0.44
*RPL36AL*		0.73**	0.34	0.35
*EIF5A*		0.36	0.62*	
*SLC9A1*			0.47	
*RPS27A*		0.40	0.45	
*MCL1*	0.43	0.38		
*MAPK3*	0.53			
*RPS18*	0.32			
*JAK3*	0.32			

### Predictive ability of the genes

**Figure 6:**
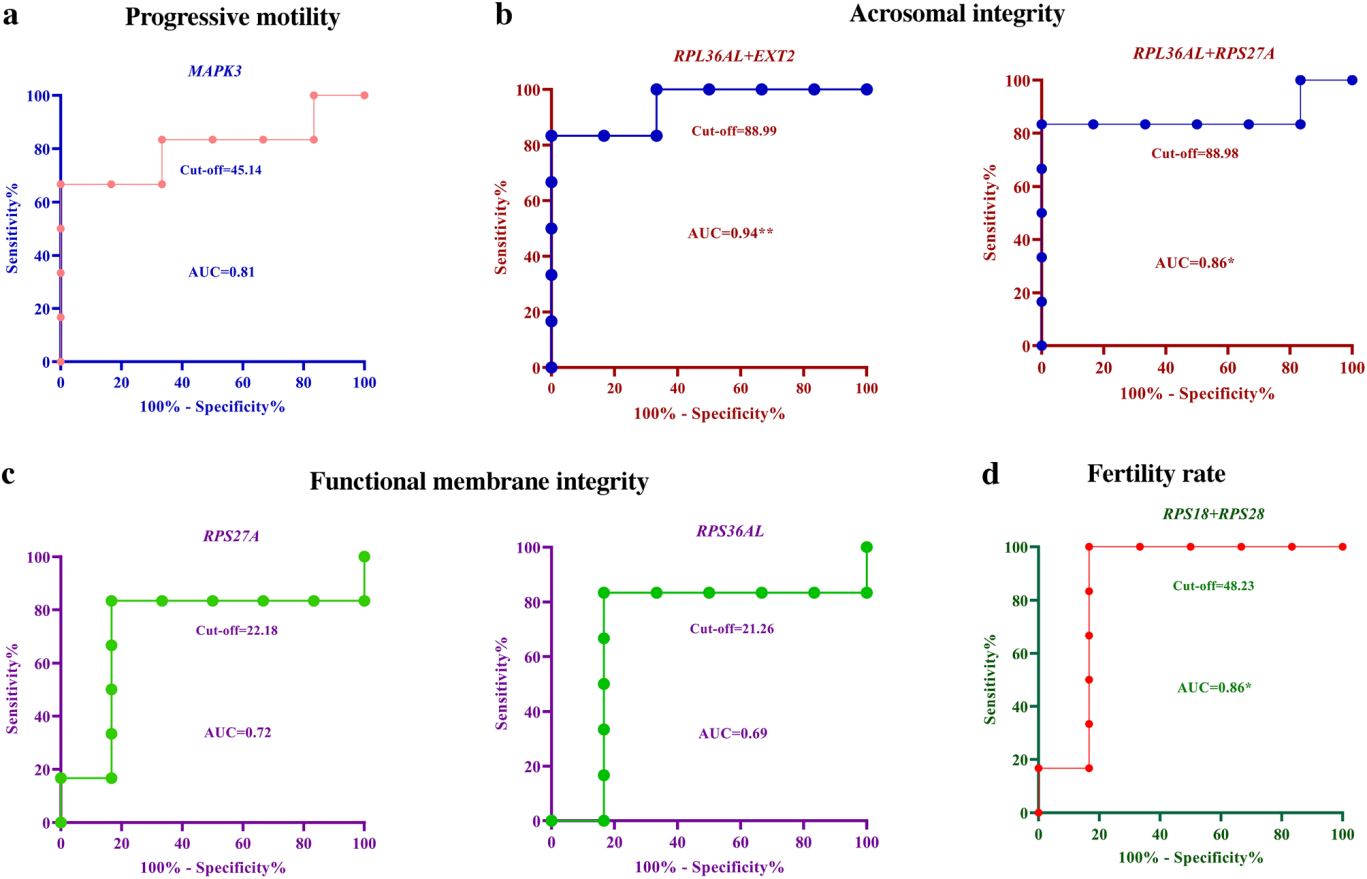
Receiver operating characteristic curve for the ability of the genes in predicting sperm functions and fertility rate. Univariate and multivariate analyses performed with the hub genes have a maximum prediction accuracy. The genes *MAPK3*, *RPL36AL, RPS27A* and *RPS28* can influence sperm progressive motility (**a**), acrosome integrity (**b**), functional membrane integrity (**c**), and fertility rates, respectively. For fertility prediction, the combination model of *RPS18* + *RPS28* had a maximum sensitivity of 100%, specificity of 83.33% and likelihood ratio of 6 (**d**).

ROC analysis of the expression levels of the single genes *MAPK3* (PM) (Fig. 6a), *EXT2* and *RPS27A* (AI) and RPS28 (FR) showed a sensitivity of 66.67%, specificity of 83.33% and likelihood ratio of 4. Similarly, the expression levels of *RPS27A* and *RPL36AL* (FMI) (Fig. 6c) and *RPS28* (FR) had a maximum sensitivity of 83.33%, specificity of 83.33% and likelihood ratio of 5. These findings suggest that these genes individually influence sperm functions and the FR.

Multiple regression analysis for the prediction of FR revealed that the combination model *RPS18* + *RPS28* had a maximum sensitivity of 100%, specificity of 83.33% and likelihood ratio of 6 (Fig 6d). The prediction models *RPS18* + *RPS28* (FR), *RPL36AL* + *EXT2* (AI) and *RPL36AL* + *RPS27A* (AI) (Fig. 6b) had a sensitivity of 83.33%, specificity of 83.33% and likelihood ratio of 5.0. All the four models were significant (*p* < 0.05) in the prediction of sperm functions and FR.

## Discussion

In the present study, hub genes influencing sperm functions and fertility rate were identified and validated. In PM sperm, the cellular response to the oxygen-containing compound process was the top enriched process in the up-regulated genes. Reactive oxygen species (ROS) are inherently generated by sperm during metabolic activities and capacitation process^12^. Excess ROS production decreases sperm motility by affecting the contractile apparatus of the flagellum^13^ and the defense response of the sperm against ROS in terms of the total antioxidant capacity is essential for sustaining total as well as progressive motility^14^. Furthermore, tyrosine kinase activity was enriched in the identified hub genes of PM sperm. Tyrosine kinases have been proved to be crucial for the sperm motility and hyperactivation^15^. In particular, *MAPK3*, known as *ERK1*, stimulates PM and hyperactivated motility in ejaculated sperm^16^. Additionally, AKAP4 protein is a substrate for *ERK1* and phosphorylation of *AKAP4* is crucial for the PM of sperm^17^. These results indicate an important role of the hub gene *MAPK3* in regulating PM. ROC analysis of the present study revealed that the expression level of single gene *MAPK3* alone had the maximum accuracy (66.67%) in predicting the semen samples with high percentages of PM sperm. These findings corroborate the important role of the identified hub gene, *MAPK3,* in regulating sperm PM. Response to lipids is an enriched process associated with downregulated genes. Lipid composition of sperm changes as it exits the male reproductive tract and when it enters the female reproductive tract. The change in composition is inevitable for a sperm to perform its functions like membrane integrity, capacitation, and acrosome reaction^18^. Thus, the high PM sperm is responding less to the lipid changes signifying the adaptation of high PM sperm to the changing lipids.

The enrichment of lipid metabolic processes in semen samples with a high percentage of intact acrosome suggests that lipid metabolism is crucial for maintaining AI. Recent literature suggests that the success of the male reproductive process, sperm motility, capacitation, acrosomal reaction and fusion of sperm and egg depends on the homeostasis of sperm lipids^18^. Sperm also utilize long-chain fatty acids for energy production^19^. Interestingly, knockout studies of lipid metabolism genes such as *Tysnd1*^20^ and *Fads*^21^ resulted in altered plasmalogen and unsaturated fatty acid levels thereby leading to incomplete acrosome formation and failure of acrosome formation, respectively. In the good AI group, the hub genes were involved in apoptosis. *MCL1,* a pro-survival factor, is required for the development and homeostasis of any tissue, and *MCL1* knockout mice are sterile with no mature sperm in the epididymis. The presence of *MCL1* in the good AI group suggests that the gene may improve acrosome integrity by regulating anti-apoptotic process^22^. The phosphorylation of kinases such as *JAK3* and *MAPK3* is required for capacitation and acrosome reaction^23^. Although there is no direct evidence of the functional roles of up-regulated genes (*RPS27A, RPL36AL* and *EXT2*) in regulating AI in the present study, we speculate that these genes have a potential role in maintaining AI. Importantly, the multivariate models *EXT2* + *RPL36AL* or *RPL36AL* + *RPS27A* had the maximum accuracy (83.3%) in predicting semen samples with good AI. Since the sperm are matured cells, down-regulation of the cell cycle genes in the high AI group indicates that these genes might have been translated into protein during spermatogenesis or these transcripts might not have been utilized by the low AI group and thus resulted in the poor acrosome quality. However, the exact function of these genes in buffalo sperm must be elucidated.

Glucose metabolism and fertilization were up-regulated in the FMI group. Sperm is the most differentiated yet metabolically active cell type and mainly utilizes glucose, fructose as a fuel source. Glucose metabolism is essential for sperm functions such as motility and fertilization events. In diabetic human males, altered glucose metabolism affects epigenetic dysregulation, leading to detrimental effects on sperm functional attributes and male fertility^24^. Bull sperm depends on oxidative phosphorylation for capacitation and binding to the oocyte, as the oviduct has a low concentration of glucose^25,26^. It has been reported that glycolytic enzymes are in the fibrous sheath of the flagellum^27^. Due to the poor metabolism of glucose in sperm, homeostasis is disturbed and ultimately affects the sperm membrane and acrosomal integrity^28^. Protein localization to the membrane (FDR = 1.11E−13) and translation (FDR = 2.2E−16) were the enriched processes of the hub genes in the good FMI group. EIF5A gene deletion leads to alterations in cell membrane integrity by affecting the PKC/WSC cell wall integrity pathway^29^ in yeast. Hypusinated EIF5A transports a subset of mRNA from the nucleus to the ribosome for translation^30^. The presence of ribosomal transcripts (*RPS36AL* and *RPS27A*) and eukaryotic translation initiation factors (*EIF5A*) in the hub genes may have led to translation enrichment. The present study indicates that the expression levels of each single gene, *RPS27A* or *RPL36AL* had maximum accuracy (83.33%) for predicting FMI. The study revealed down-regulation of RNA splicing process in high FMI group. Sperm splicing events regulate sperm functions and bull fertility^31^. Hence, the down-regulation of splicing might be because the associated transcripts might have been translated to proteins during spermatogenesis in the high FMI group.

The number of up-regulated genes in the FR group was comparatively lower and that may be the reason for not observing any significant enrichment. Previous research in cattle from our lab revealed that among the differentially expressed genes, the genes linked with the bull fertility rate were closely associated with the genes regulating functional membrane integrity, and acrosome integrity^1^. Likewise, in the present study translation was observed to be the top enriched biological process of the hub genes in FR as well as in the FMI group. The hub genes of this process include RPLs and RPSs subunits, indicating ribosomal heterogeneity between good FMI and FR. Previously, differentially expressed ribosomal transcript markers have been observed in immotile sperm^32^, abnormal morphology^33^, conception^34^, and fecundity^35^. Along with that, in the present study, ribosomal subunit genes, such as *RPLP0*, *RPS18*, and *RPL36AL,* were upregulated in the good FR group. The present study also suggests that the combination model *RPS28* + *RPS18* had the highest accuracy (83.33%) in predicting FR. Earlier studies from our laboratory have also identified a strong positive association between RPLs and FR^34^. In boar testis, expression levels of RPL18 have been positively associated with pubertal development^36^. These ribosomal proteins may aid in the translation of sperm RNAs after fertilization or may be involved in the translation of sperm mitochondrial genes or may be involved in processes other than translation. However, further investigation is required to understand the role of ribosomal gene families in predicting sperm fertility.

In the present study, there was a heterogeneity in the expression of ribosomal large and small subunit protein genes of mature buffalo sperm. Ribosomal heterogeneity in sperm is not yet elucidated completely and hence further research in this area will identify the gene regulations coded by the ribosome heterogeneity^37^.

Clearly, the only overlapping gene, *EXT2,* is a glycosyltransferase up-regulated in all four groups of DESeq2 analysis and was validated in the AI and FR groups. Quantitative dot blot analysis of the representative hub genes indicates that the protein levels of EXT2 is not in trend with the RNA-seq and RT-qPCR findings. The finding suggest that the available transcripts would have been translated to protein in the sperm. This glycosyltransferase is involved in the binding of the sperm to the ZP3 protein of an egg during fertilization^38^ and initiates acrosomal exocytosis. The differential gene and protein expression of EXT2 in FR group implies that *EXT2* may be involved in the maintenance of acrosomal integrity and acrosomal exocytosis during sperm binding to zona, thereby, regulating the fertility of a bull.

Though ROC analysis revealed that the expression level of a single gene is sufficient for predicting PM and FMI status, the two-genes combination models are required to achieve maximum accuracy in the FR and AI groups. These results indicate that a correct combination of genes is required to achieve maximum prediction accuracy^39^. However, detailed studies are required to elucidate the role of these novel genes in regulating sperm functions and FR.

**Figure 7:**
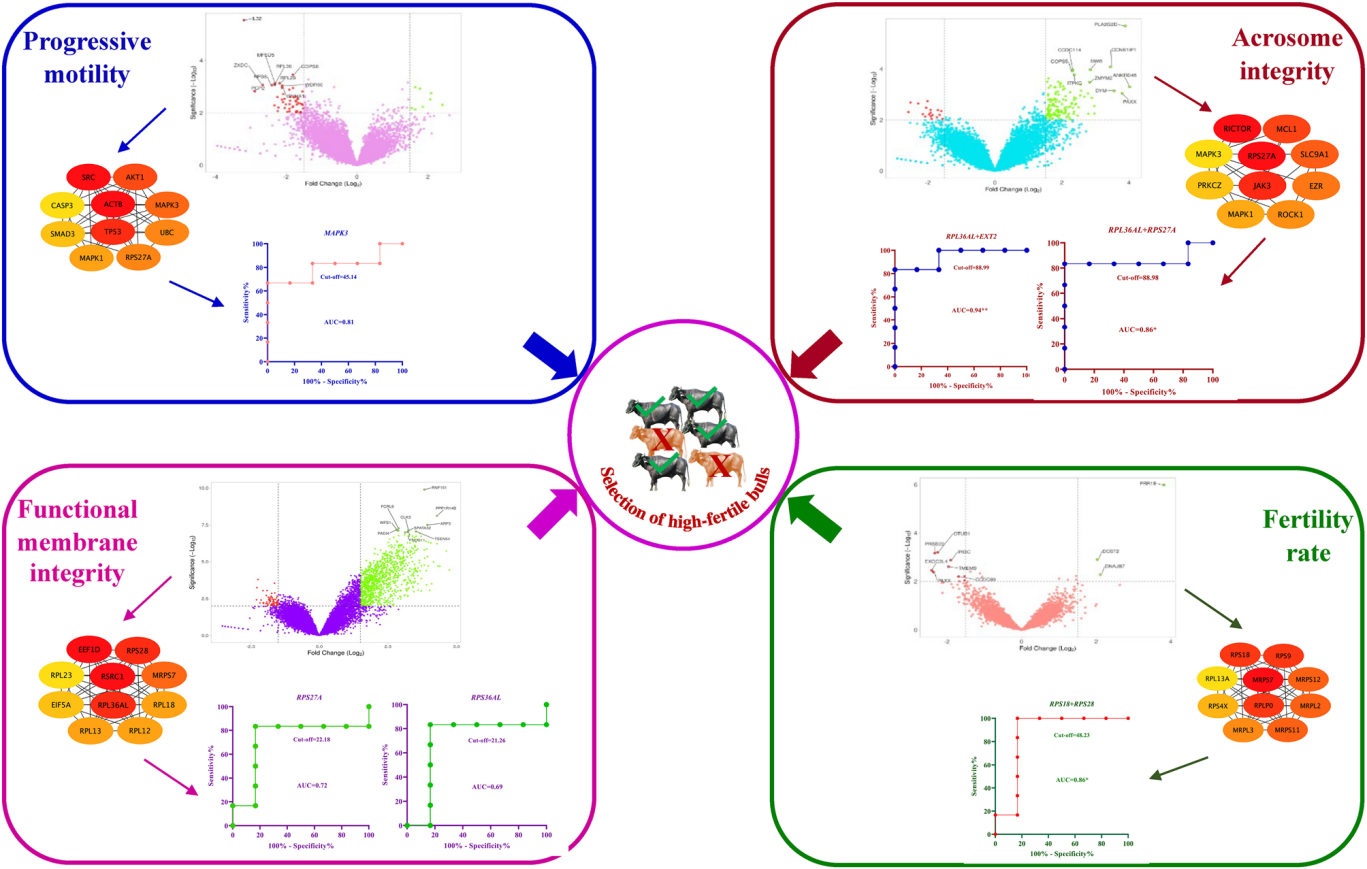
Graphical abstract depicting the identification of hub genes in sperm fertilization competence. The differentially expressed genes and their corresponding hub genes were identified in the bulls of different fertilization competence. The hub genes were validated using RT-qPCR and the receiver operating characteristic curve analysis identified *MAPK3* (PM), *RPL36AL* + *RPS27A* or *RPL36AL* + *EXT2* (AI)*; RPL36AL* or *RPS27A* (FMI) and *RPS18* + *RPS28* (FR) can be used to select the high fertile bulls.

Thus, the expression levels of *MAPK3* (PM), *RPL36AL* (AI), *EIF5A* (FMI) as well as *RPLP0* and *RPS28* (FR) in good-quality semen indicate that these genes can be used to diagnose the semen quality and fertility status of bulls (Fig. 7). In particular, the combination model *RPS18* + *RPS28* can be helpful in predicting the fertility status of bulls. Importantly, they can serve as gene markers to identify the high-fertile bulls for the breeding program. These hub genes can also be of drug targets for the improvement of sperm functions and bull FR.

## Materials and methods

**Figure 8:**
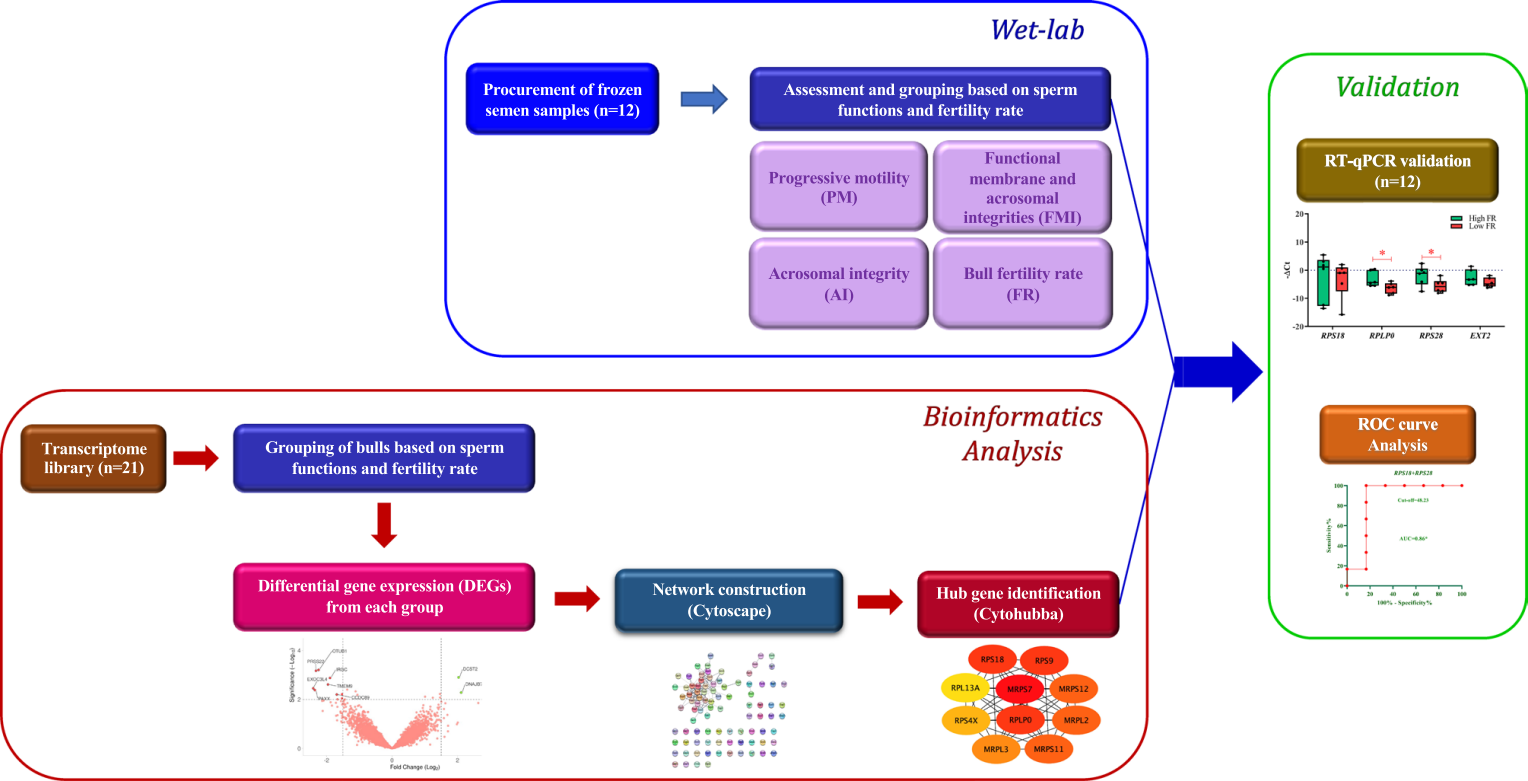
Methodological overview of the hub gene identification for bull fertility prediction. The methods include sperm function assessment, grouping based on sperm functions and FR, identification of differentially expressed genes in each group, up-regulated genes network construction for each group, identifying the intersection of all networks, identification of clusters, identification of hub genes, validation using RT-qPCR and receiver operating characteristic curve analysis.

In brief, the methodology includes the procurement of frozen semen samples, assessment of sperm functions, RNA isolation and library preparation for RT-qPCR. Bioinformatic analysis of the transcriptome data includes the identification of differentially expressed genes, enrichment analysis and identification of the hub genes. The identified hub genes are validated using RT-qPCR and the association of the genes to the sperm functions and fertility rate was determined using correlation analysis (Fig. 8). The expression levels of the genes were used to diagnose the sperm functions and fertility rate status of the bulls.

### Sample details

#### Animal ethics

All the experiments were conducted as per the approval of the Institute Animal Ethics Committee (IAEC approval vide: NIANP/IAEC/1/2020/12). All the methods were performed in accordance with the relevant guidelines and regulations.

#### Sample procurement

Frozen semen samples (n = 12) of the Murrah buffalo (*Bubalus bubalis*) bulls were purchased from ICAR-Central Institute for Research on Buffaloes (ICAR-CIRB), Haryana, India and stored in liquid nitrogen (− 196 °C) until further analyses.

### Sperm functions

Frozen semen samples were thawed at 37 °C for 30 s in a water bath and sperm functions such as progressive motility (PM), acrosomal integrity (AI), and functional membrane integrity (FMI) were evaluated for each of the ejaculates (at least 2 ejaculates per animal)^40–42^.

#### Progressive motility

Progressive motility and other sperm kinematics were analyzed using a computer-assisted semen analyzer (CASA, Sperm Class Analyzer, version 6.4, Microptic, Spain). The frozen semen sample was thawed at 37 °C for 30 s in a water bath and diluted in Tris buffer (1:5). The diluted sample (10 μl) was then placed on the prewarmed (37 °C) glass slide and covered with an 18 × 18 mm coverslip. The sample was then analyzed for sperm kinematics including PM, total motility, velocities, wobble, etc. using a negative phase-contrast microscope (Nikon Eclipse 50, Nikon, Japan). The images were captured at the rate of 25 frames/s from 10 homogenous fields per ejaculate using the configuration settings: cell size: range 10–70 μm^2^; PM: sperm with a speed of >50μm/sec and >70% straightness (STR).

#### Functional membrane integrity and acrosomal integrity

Both the FMI and AI were evaluated using a single test hypoosmotic swelling- Giemsa (HOS-G) test. Thawed (37 °C) semen sample (50 μl) was added to 450 μl of hypoosmotic (100 mOsm) and isoosmotic solutions (300 mOsm) maintained at 37 °C and then incubated for 30 min. After the incubation, 10 μl of the solution from each medium was smeared onto a clean glass slide, air-dried, fixed and then stained using Giemsa. Sperm were classified into four populations: Host positive and acrosome positive (HPAP), Host positive and acrosome negative (HPAN), Host negative and acrosome positive (HNAP) and Host negative and acrosome negative (HNAN). Sperm with hairpin bent tails were considered Host positive otherwise considered Host negative. Sperm with intact acrosomal membranes were considered acrosome positive and otherwise considered acrosome negative. The actual population of both functional membrane and acrosome intact sperm (FMI) was calculated by subtracting the HPAP positive population in 300 mOsm from the HPAP of the hypoosmotic (150 mOsm) solution. Sperm population of HPAP and HNAP in 300 mOsm were added together for calculating the percentage of acrosomal intact (AI) sperm. A minimum of 200 sperm were counted using 100 × objective under the phase-contrast microscope (Nikon Eclipse 80i, Nikon, Japan). An average value for the two ejaculates per bull was calculated and considered for further statistical analysis.

Based on the group average for sperm functions and FR, the bulls were divided into two groups (n = 6, good and poor). All the functions and FRs differ significantly (*p* < 0.05) between the groups.

#### Bull fertility rate

The field fertility data for each bull were obtained from the semen bank. The fertility rate for each bull was calculated based on at least 500 inseminations per bull and verified pregnancy after 60 days of insemination.

### Bioinformatics analysis

#### Differentially expressed genes

Differentially expressed genes (*p* < 0.05 and log_2_ fold change > 1) from the PM, AI, FMI and FR groups were analyzed from the sperm RNA-seq data (n = 21, Reproductive Physiology Laboratory, ICAR-NIANP, Adugodi, Bengaluru) of buffalo bulls. The RNA-seq datasets analyzed during the current study have been submitted to the NCBI database SRA Bioproject PRJNA803987. Due to proprietary reasons the data are not publicly available.

#### Enrichment analysis

Enrichment analysis of both up-regulated and down-regulated genes in each group was performed the using Gene-set enrichment analysis tool (GSEA, version 4.2.3) with the GSEA Preranked module: 1000 permutations; the minimum and the maximum size to exclude gene sets were set to 2 and 500, respectively. Enriched processes and their corresponding normalized enrichment score (NES) were obtained from GSEA, whereas the redundant processes with broad meaning were eliminated.

#### Construction of gene network

The network corresponding to both up-regulated and down-regulated genes from each group was imported into the Cytoscape (version 3.8.2) using the network import module with the STRING database as the background data source. The network with default combined interaction scores greater than 0.4 were imported with 10 additional interactors for subsequent analysis. Also, an intersection and union of all four networks were constructed using the merge option in the Cytoscape.

#### Module analysis

A molecular complex detection (MCODE) tool from the Cytoscape plug-in was used to identify the densely connected clusters with the following default parameters: score cut-off = 5; K-score = 2; node score cut-off = 0.2; max depth from seed = 100. Enrichment of the top cluster was studied using the STRING tool (version 11.5) and the significant values were denoted as False discovery rate (FDR).

#### Analysis of hub-genes

The top 10 hub genes were identified from the top cluster of each group through the CytoHubba plugin of Cytoscape. The hub genes were identified using each of the topological analysis methods such as Betweenness, BottleNeck, Closeness, Clustering Co-efficient, Degree, Density of Maximum Neighborhood Component (DMNC), EcCentricity, Edge Percolated Component (EPC), Maximal Clique Centrality (MCC), Maximum Neighborhood Component (MNC), Radiality, and Stress. The common 10 genes from each of these topological analysis methods were shortlisted as the key hub genes in our study since the overlap of all methods gives the most probable hub genes^43^. From the shortlisted hub genes, the genes with the highest average FPKM from each group were chosen for RT-qPCR validation.

### RNA isolation and cDNA synthesis

Buffalo sperm RNA was isolated using the previously established protocol established in our laboratory^44^. Briefly, frozen semen samples were washed using 50% Bovipure (Nidacon, Sweden) solution. After washing, 40 × 10^6^ sperm per sample were taken and lysed with a double lysis method followed by extraction using a silica membrane-based column (PureLink RNA mini kit, Invitrogen, USA). The extracted total RNA was subjected to DNase treatment (Turbo DNA-free kit, Ambion, USA) to remove the contaminating genomic DNA. The total RNA concentration was measured using a fluorometer (Qubit 4.0, Invitrogen, USA) and RNA quality was measured using a spectrophotometer (NanoDrop ND-1000, Thermo Scientific, USA). The RNA integrity was estimated using Bioanalyzer (2100 Bioanalyzer, Agilent Technologies, USA).

Total sperm RNA (100 ng) was used for library preparation and amplification using the NEBNext Ultra II Directional RNA library kit (New England Biolabs, USA). Precisely, total RNA was reverse transcribed to cDNA using random hexamers. The complementary strand was synthesized using DNA polymerase-I, subsequently amplified for 15 PCR cycles and used for gene expression studies.

### Gene expression studies

The expression levels of the hub genes identified from the CytoHubba tool were quantified using the RT-qPCR (StepOnePlus, Applied Biosystems, USA). Each RT-qPCR reaction consists of an equal concentration of amplified cDNA from each bull, 1X SYBR Green Mastermix with ROX (TB Green Premix Ex Taq II, Takara Bio, Japan) and 125 nM each of forward and reverse primers (Table 6). The absence of RNA from other contaminating cells was ensured in each sperm sample with the cell-specific primers for *KIT* (germ cells), *CDH1* (epithelial cell) and *PTPRC* (leukocytes) (Supplementary Fig. S4). The PCR cycle conditions were 95 °C for 30 s, 40 cycles of 95 °C for 5 s and 60 °C for 1 min followed by the default melt curve settings. The data were acquired and analyzed in the StepOne software (v2.2.2). Relative gene expression levels were calculated using the 2^−ΔΔCt^ method^45^ using *GAPDHS* as the housekeeping gene. The PCR products were also checked using 2% agarose gel electrophoresis (Supplementary Fig. S4).

**Table 6:** List of primers used for gene expression studies and RNA quality control. The primers *PRM1* and *GPX4, PTPRC, CDH1* and *KIT* were used as quality control to check DNA, leukocytes, germ cell and epithelial cell contamination, respectively in the isolated RNA. *GAPDHS* was used as a housekeeping gene and the other primers were used for gene expression studies.

S. no.	Gene name	Primer sequence 5′ to 3′	Product size (bp)	NCBI accession number
1	*PRM1*	F-ATGGCCAGATACCAATGCT	224	From our database
		R-GTGGCATGTTCAAGATGTGG		
2	*GPX4*	F-AATGTGGCCTCGCAATGAGG	164	XM_025292793.2
		R-CCAGCGGCGAACTCTTTGAT		
3	*PTPRC*	F-TTCAGAAGGACGCATGCTGT	137	XM_025285310.1
		R-GGTGGGGTAGAGTTTCCTGC		
4	*CDH1*	F-CTGCATTCCTGGCTTTGGTG	171	XM_006047636.2
		R-GTAAGCACGCCATCTGTGTG		
5	*KIT*	F-GAATAGCTGGCATCAGGGTG	224	NM_001290952.1
		R-CAGATCCACATTCTCTCCATC		
6	*GAPDHS*	F-CAGATGCACCCATGTTTGTC	175	XM_006068806.3
		R-GCCGTGTAGGAATGGACTGT		
7	*MCL1*	F-TTTCTTTTGGTGCGTTTGTG	202	XM_006058113.3
		R-AAAGCCAGCAGCACATTTCT		
8	*MAPK3*	F-ACAGTCTCTGCCCTCCAAGA	245	XM_045164289.1
		R-GCTCCTTCAGTCGTTCCTTG		
9	*SLC9A1*	F-TTCCTGGACCACCTTCTGAC	195	XM_006055479.4
		R-TTCCACCAGCTCGATAGCTT		
10	*JAK3*	F-AATTCCAGTGCCAGCTGAGT	233	XM_025293831.3
		R-TGACACTTGGCTCATCAAGC		
11	*RPS18*	F-AAAATTGCCTTTGCCATCAC	212	XM_044944055.2
		R-TATTTCCCGTCCTTCACGTC		
12	*RPL36AL*	F-GCCTCACAAAGTGACCCAGT	207	XM_006063136.4
		R-CACCTCTTAATGGCCAGCAT		
13	*RSRC1*	F-ATCTTCAGCCTCGCTCACAT	170	XM_006079554.4
		R-TGATCGGGACCTTCTTGTTC		
14	*EIF5A*	F-ACTTCTAAGACCGGCAAGCA	147	XM_006060160.3
		R-GCCAATCAGCTGAAAATCGT		
15	*EXT2*	F-TCATCCCGAGAATGAAGACC	236	XM_045162919.1
		R-GTCGAAACATGTGTGCATCC		
16	*RPLP0*	F-CAGCAGGTGTTTGACAATGG	233	XM_006052971.4
		R-GCCTTGACCTTTTCAGCAAG		
17	*RPS28*	F-CCGAAACACGTGACTCCTCT	190	XM_045166697.1
		R-CCTTTCACGTTTCGGATGAT		
18	*ACTB*	F-GCGCAAGTACTCTGTGTGGA	191	XM_025274489.3
		R-ATCTCGTTTTCTGCGCAAGT		
19	*RPS27A*	F-GCCAAGATGCAGATTTTCGT	167	XM_006043249.4
		R-CGTCCATCTTCCAGTTGCTT		

### Quantitative dot blot

Quantitative dot blot was performed with minor modifications^46^. Differential protein estimation was done using new set of bulls (n = 13). Total sperm lysate (1 µg/bull) from high (n = 8) and low (n = 5) fertility groups were applied directly to the nitrocellulose membrane. The membrane was allowed to dry at 37 °C for 30 min and blocked with 5% bovine serum albumin in TBST (19.97 mM Tris–HCl, 136.89 mM NaCl, pH 7.6 plus 0.1% Tween-20) and incubated at 37 °C for 1 h. Then, the primary antibodies, rabbit anti-RPS18 polyclonal antibody (A11687, ABclonal, USA), rabbit anti-EXT2 polyclonal antibody (A1973, ABclonal, USA), and rabbit anti-RPLP0 polyclonal antibody (PA5-41717, ThermoFisher, USA) were added to the respective membrane and incubated at 37 °C for 120 min. Rabbit anti-GAPDHS polyclonal antibody (A10471, ABclonal, USA) was used as housekeeping protein. Then the membrane was washed thrice for 5 min each in TBST buffer. Later, the secondary antibody was added to the membrane and incubated at 37 °C for 60 min. After incubation, the membrane was washed in TBST for three times. The image was developed using ECL substrate solution (Immobilon ECL Ultra substrate, Thermofisher, USA) and the signals were captured by chemi-documentation system (iChemi XR, Syngene, UK). The relative abundance of the protein between high and low fertility group was calculated based on densitometric analysis using GENE TOOLS software (Syngene, UK).

### Receiver operating characteristic (ROC) curve analysis

The fertility predictive value of the validated genes was evaluated using ROC curve analysis. First, the predictive power of individual genes was analyzed using univariate regression analysis. Subsequently, multivariate regression analysis was performed by combining the expression levels of these genes. The linear regression models were developed by employing PM, AI, FMI and FR as independent variables and ΔCt of the genes as the dependent variable. The ROC analysis was performed to assess the sensitivity (%), specificity (%), accuracy (%) and diagnostic efficiency (%) of the univariate and multivariate regression models at the chosen cut-off with a maximum likelihood ratio for classifying the bulls into their respective good or poor categories^34^.

### Statistical analysis

The sperm functions were subjected to statistical analysis using the IBM SPSS statistics 20 and GraphPad Prism 6. All the sperm function data were normally distributed and hence the Student’s *t*-test was used for calculating the significance between the groups. The correlation between the gene expression levels and the functional parameters was analyzed using the Pearson correlation coefficient. The correlation (*r*) values of < 0.1, 0.1 to 0.3, 0.3 to 0.5 and > 0.5 were considered trivial, small to medium, medium to large and large to very large, respectively^47^. All the values were presented as mean ± SEM and the significance is set at *p* < 0.05.

### Supplementary Information

Supplementary Figures.

## Data Availability

RNA-seq data are not generated in this study. The data used for the present study are not publicly available due to proprietary nature but are available from the corresponding author on reasonable request.
