# Supplementary material for: Identification of hub genes and their expression profiling for predicting buffalo (Bubalus bubalis) semen quality and fertility
Source: Sci Rep. 2023 Dec 13;13:22126. doi: 10.1038/s41598-023-48925-5 (PMC10719284; doi:10.1038/s41598-023-48925-5)
Supplement: Supplementary file 1 — Supplementary Figures. [file 41598_2023_48925_MOESM1_ESM.pdf]

Supplementary Information

Identification of hub genes and their expression profiling for predicting buffalo *(Bubalus bubalis)* semen quality and fertility

Divakar Swathi[a,b], Laxman Ramya[a], Santhanahalli Siddalingappa Archana[a], Balaganur Krishnappa[a], Bala Krishnan Binsila[a] and Sellappan Selvaraju[a,*]

[a]*Reproductive Physiology Laboratory, Animal Physiology Division, ICAR-National Institute of Animal Nutrition and Physiology, Adugodi, Bengaluru-560030, India.*

[b] *Department of Biotechnology, Jain University, Bengaluru, 560001, India.*

**Corresponding author, Sellappan Selvaraju, ICAR-National Fellow, Reproductive Physiology Laboratory, ICAR- National Institute of Animal Nutrition and Physiology, Bengaluru, India. E-mail: Selvaraju.S@icar.gov.in*

Supplementary Figure S1

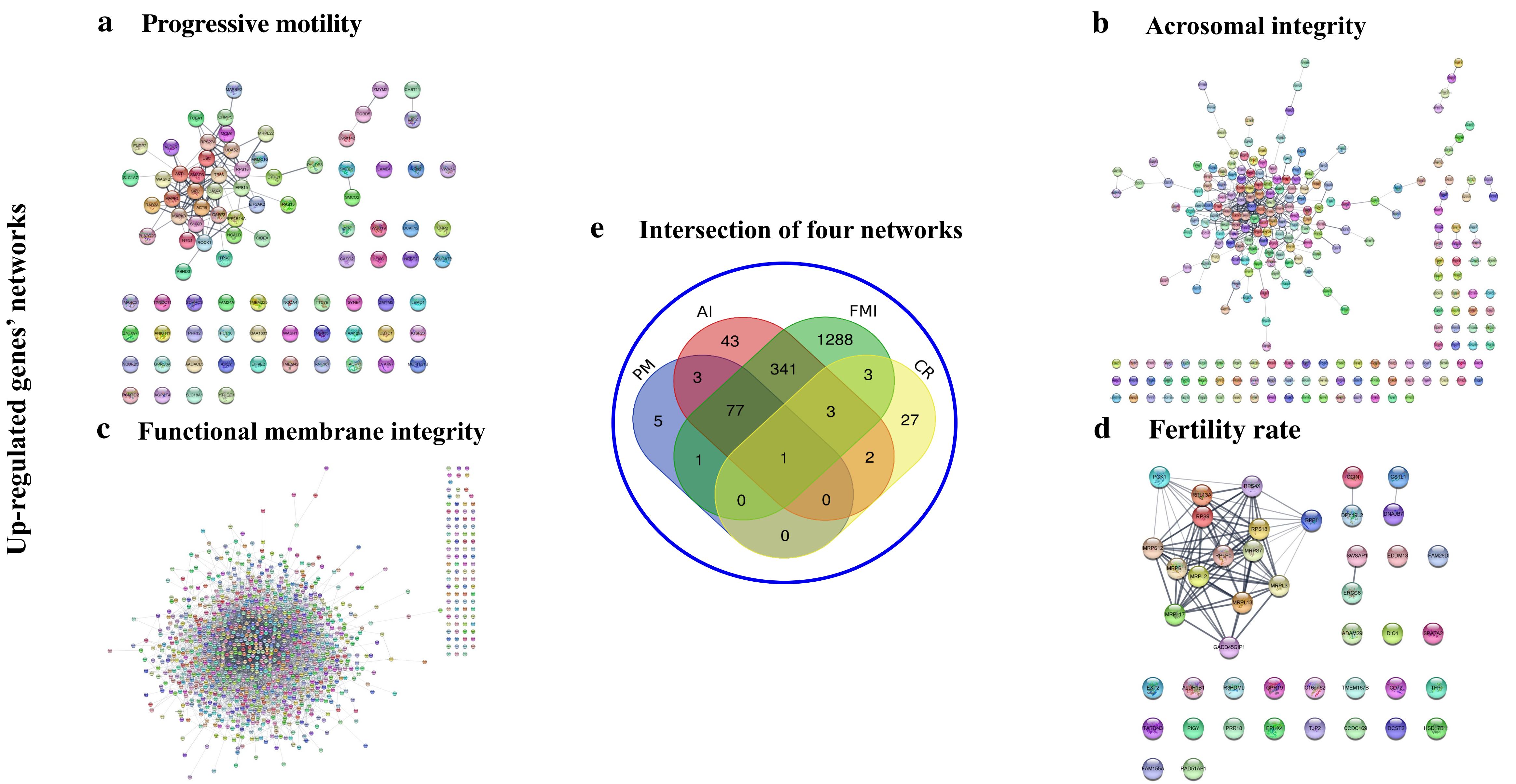

Supplementary Fig. S1: Identification of networks from the up-regulated genes based on sperm functions and fertility rate. Gene network of up-regulated genes from progressive motility **(a)**, acrosomal integrity (**b**), functional membrane integrity (**c**) and fertility rate (**d**) visualized in the Cytoscape tool. The intersection of all four networks resulted in a single node which is *EXT2* **(e)**.

Supplementary Figure S2

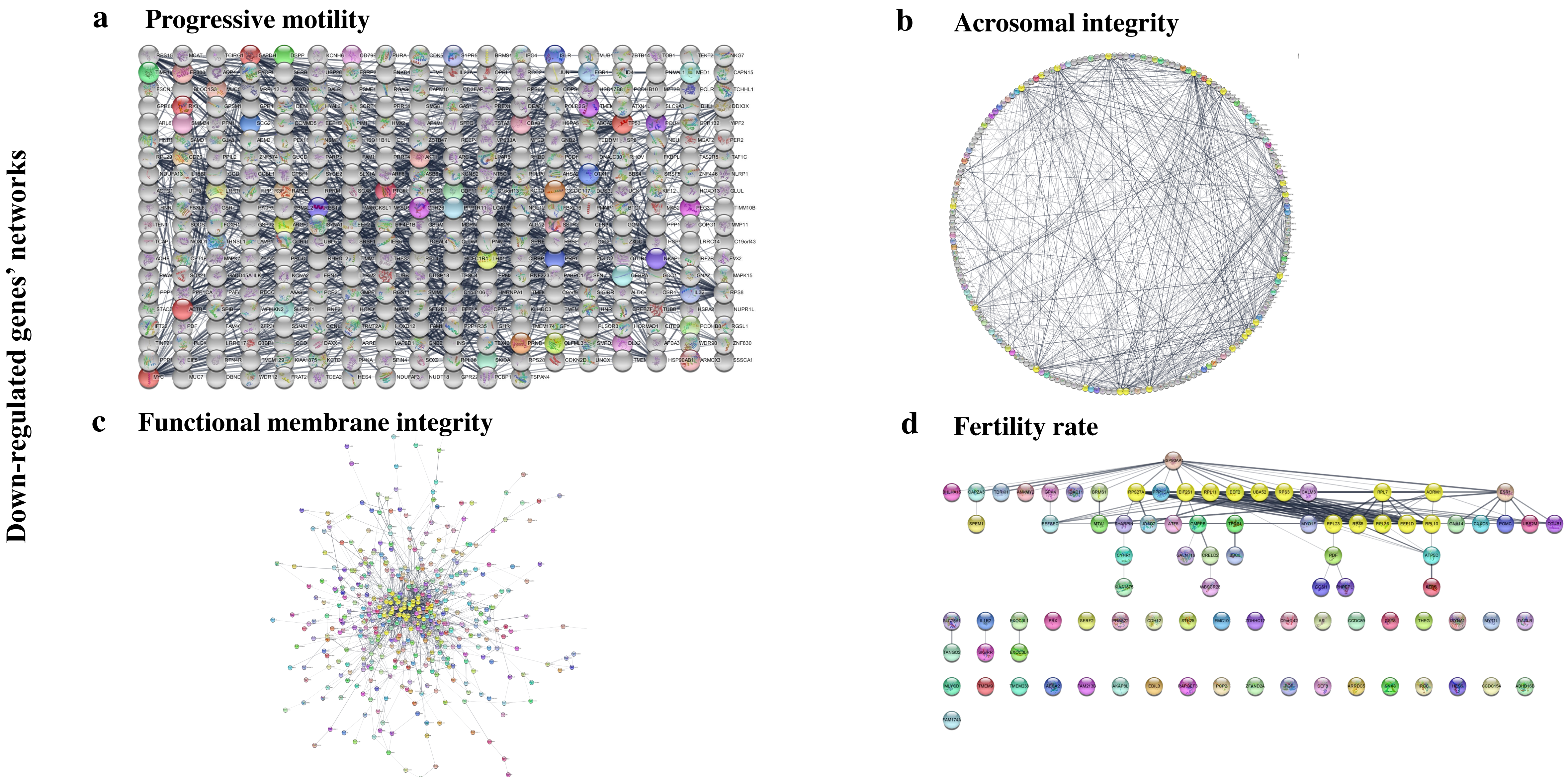

Supplementary Fig. S2: Identification of networks of the down-regulated based on sperm functions and fertility rate. Gene network of down-regulated genes from progressive motility **(a)**, acrosomal integrity (**b**), functional membrane integrity (**c**) and fertility rate (**d**) visualized in the Cytoscape tool.

Supplementary Figure S3

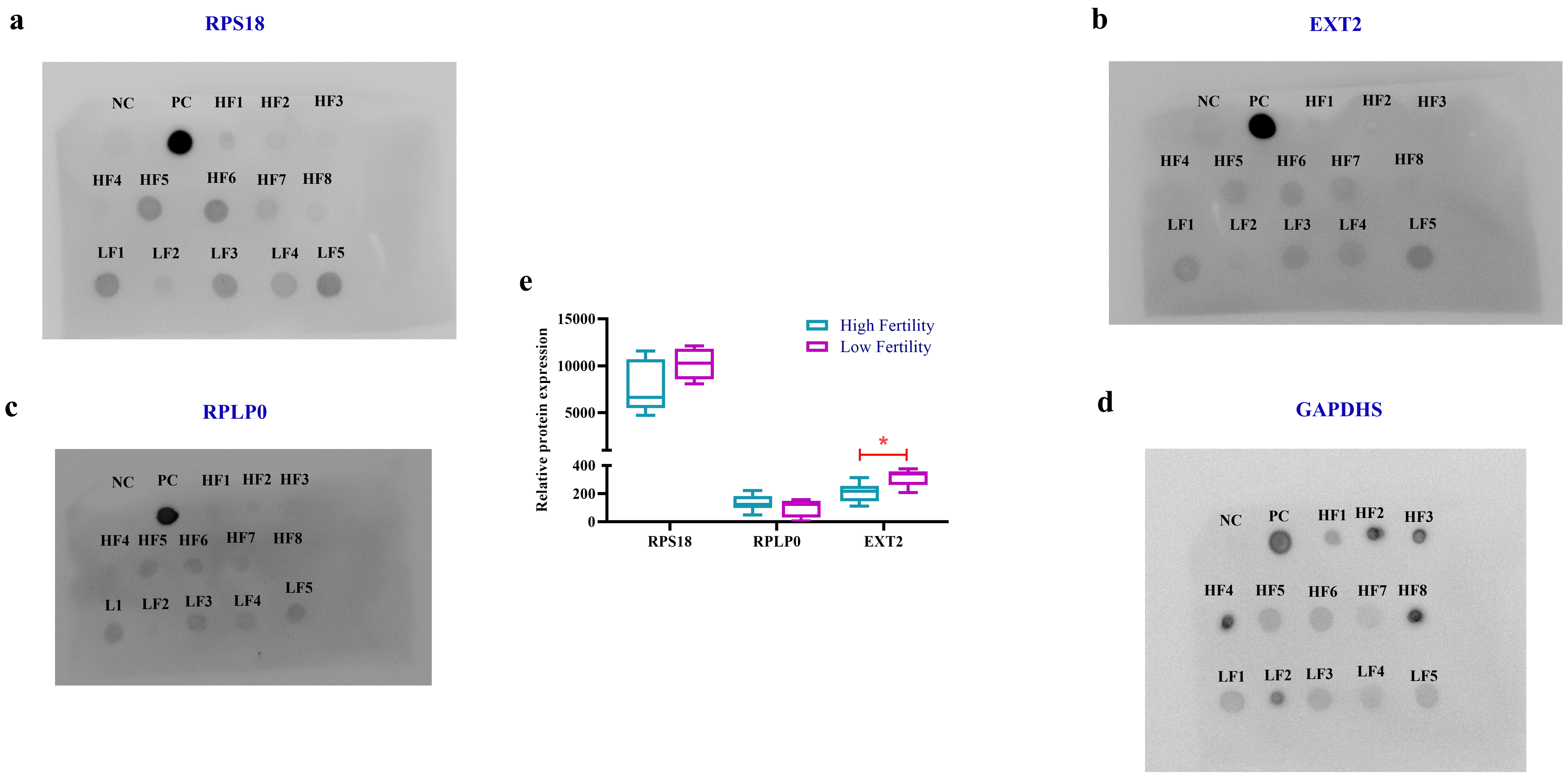

Supplementary Fig. S3: Dot blot analysis of the representative proteins of the hub genes. Dot blot images of the buffalo sperm proteins validated- RPS18 **(a)**, EXT2 **(b)**, RPLP0 **(c)** and GAPDHS **(d).** Relative expression levels of the protein were estimated by normalizing with the house keeping protein GAPDHS. The protein levels of EXT2 significantly ($p < 0.05$) varied between high and low fertility group **(e)**. HF and LF denotes High Fertility and Low Fertility; NC and PC denotes Negative and Positive controls.

Supplementary Figure S4

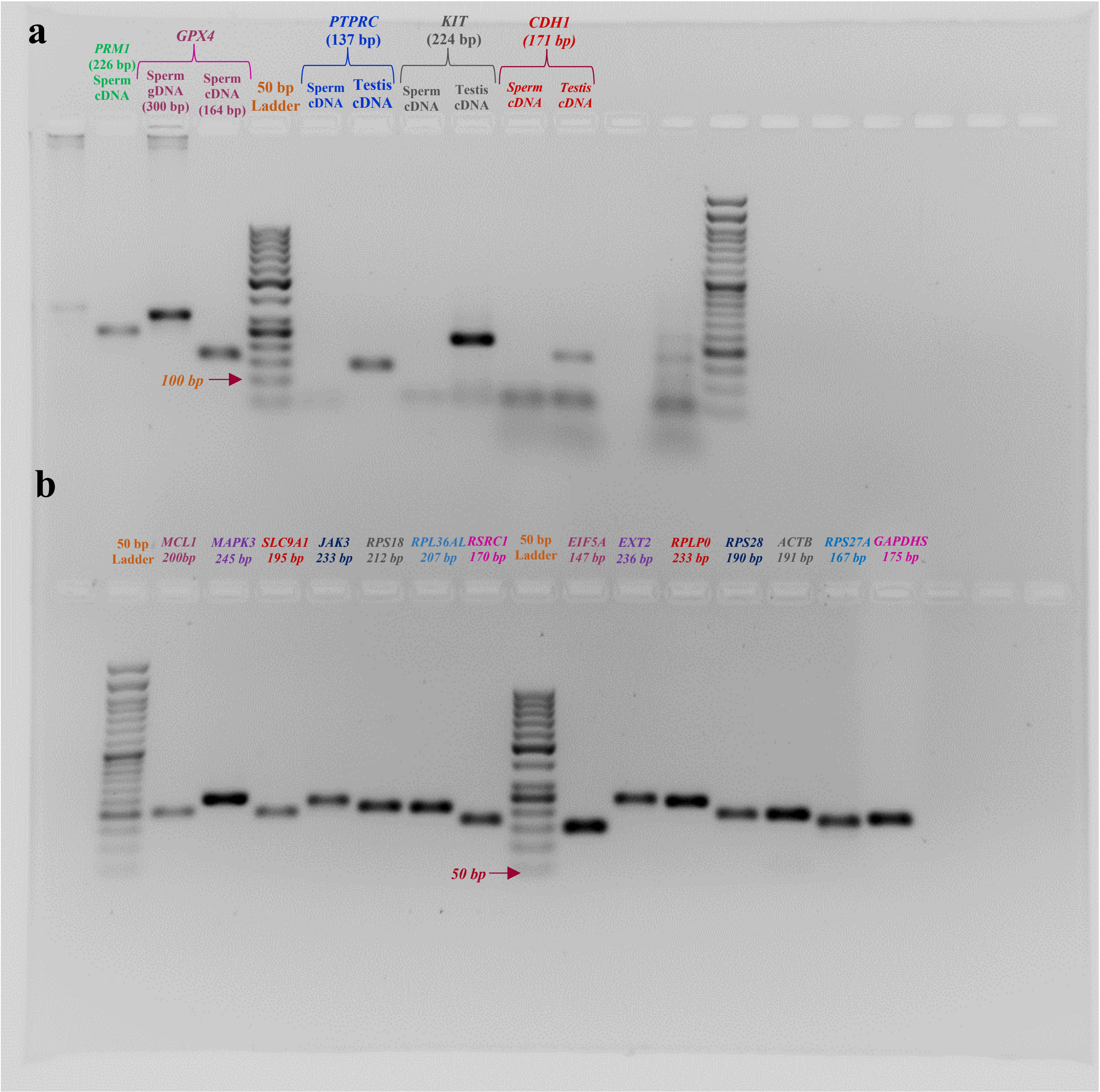

Supplementary Fig. S4: Sperm RNA quality checks and PCR amplification products of the genes used in this study. Primers for sperm RNA validation using *PRM1*; DNA contamination check using *GPX4*; leukocytes, germ cell and epithelial cell contamination checks using *PTPRC, KIT* and *CDH1*, respectively were checked in each sperm RNA before proceeding for library preparation and gene expression studies (**a**). PCR amplification products of the genes used in this study for validation and housekeeping gene *GAPDHS* were checked using 2% agarose gel electrophoresis (**b**).
